# Re-Evaluation of Phylogenetic Relationships among Species of the Mangrove Genus *Avicennia* from Indo-West Pacific Based on Multilocus Analyses

**DOI:** 10.1371/journal.pone.0164453

**Published:** 2016-10-07

**Authors:** Xinnian Li, Norman C. Duke, Yuchen Yang, Lishi Huang, Yuxiang Zhu, Zhang Zhang, Renchao Zhou, Cairong Zhong, Yelin Huang, Suhua Shi

**Affiliations:** 1 State Key Laboratory of Biocontrol and Guangdong Key Laboratory of Plant Resources, Sun Yat-sen University, Guangzhou, Guangdong, China; 2 Trop WATER, James Cook University, Townsville, Queensland, Australia; 3 Hainan Dongzhai Harbor National Nature Reserve, Haikou, Hainan, China; National Cheng Kung University, TAIWAN

## Abstract

*Avicennia* L. (Avicenniaceae), one of the most diverse mangrove genera, is distributed widely in tropical and subtropical intertidal zones worldwide. Five species of *Avicennia* in the Indo-West Pacific region have been previously described. However, their phylogenetic relationships were determined based on morphological and allozyme data. To enhance our understanding of evolutionary patterns in the clade, we carried out a molecular phylogenetic study using wide sampling and multiple loci. Our results support two monophyletic clades across all species worldwide in *Avicennia*: an Atlantic-East Pacific (AEP) lineage and an Indo-West Pacific (IWP) lineage. This split is in line with biogeographic distribution of the clade. Focusing on the IWP branch, we reconstructed a detailed phylogenetic tree based on sequences from 25 nuclear genes. The results identified three distinct subclades, (1) *A*. *rumphiana* and *A*. *alba*, (2) *A*. *officinalis* and *A*. *integra*, and (3) the *A*. *marina* complex, with high bootstrap support. The results strongly corresponded to two morphological traits in floral structure: stigma position in relation to the anthers and style length. Using Bayesian dating methods we estimated diversification of the IWP lineage was dated to late Miocene (*c*. 6.0 million years ago) and may have been driven largely by the fluctuating sea levels since that time.

## Introduction

*Avicennia* L., an important component of forests worldwide in tropical and subtropical intertidal zones [1, 2], is currently thought to be derived from within the Acanthaceae based on recent phylogenetic studies (APG III, [3, 4]), although it has been placed in Verbenaceae or Avicenniaceae in some classifications. *Avicennia* has developed several unique features, such as salt glands, aerial and prop roots and finger-like pneumatophores, and cryptoviviparous fruits with the ability to float in salt water [2]. Members of the genus often occupy newly-formed seaward mangrove habitats on tropical coasts.

*Avicennia* L. is one of the most diverse mangrove genera, comprising eight species: *A*. *germinans* (L.) Stearn, *A*. *schaueriana* Stapf & Leechm. ex Moldenke, *A*. *bicolor* Standley, *A*. *marina* (Forssk.) Vierh, *A*. *alba* Blume, *A*. *officinalis* L., *A*. *integra* N. C. Duke, and *A*. *rumphiana* Hallier f [1]. Among them, the first three are endemic to the Atlantic-East Pacific (AEP) region; the remaining five are in the Indo-West Pacific region (IWP) [1]. One of the species in the IWP, *A*. *marina*, has the broadest distribution of all mangroves, ranging from approximately 25° N to 38° S. Three infraspecific taxa within *A*. *marina* have been recognized: *A*. *marina* var. *marina*, var. *eucalyptifolia*, and var. *australasica* [1, 5]. A recent systematic revision of *Avicennia* [1] based on morphological studies [6] divided the five IWP species into three groups: (i) *A*. *marina*; (ii) *A*. *officinalis* and *A*. *integra*; and (iii) *A*. *rumphiana* and *A*. *alba* [6]. However, detailed evolutionary relationships among the species of *Avicennia*, such as chronological speciation order and divergence time, have not been clearly described so far. Evidence from molecular markers [7–9] shed some light on the patterns of population genetics and phylogeny of *Avicennia* species in the AEP region. However, so far studies of the species from IWP have been relying on morphological and allozyme data [5].

Sequencing multiple loci has been a successful strategy used to resolve phylogenetic relationships among numerous species [10, 11]. Thus, we set out to re-evaluate the phylogenetic relationships among species in *Avicennia* based on a robust approach involving wide sampling and sequencing of a number of DNA segments. Specifically, we address the following questions: (1) Do molecular data support a phylogenetic grouping based on geographical distributions of AEP and IWP lineages? (2) What are the phylogenetic relationships of the five IWP *Avicennia* species and what do they tell us about evolution of flower characters? (3) When did the IWP *Avicennia* species diversify and what was the possible driving force behind this divergence? Our results shed fresh light on the evolution of this important mangrove clade and should enable further studies of this group’s remarkable adaptation to the intertidal environment.

## Materials and Methods

### Ethics statement

*Avicennia* species used in the manuscript are not included in the China, Thailand, Indonesia, Malaysia, Bangladesh, Kenya, Australia or New Zealand official lists of endangered plants. No specific permission was required for sampling these species in this study. The fieldwork was outside protected areas or natural reserves, and no specific permission was required for these collection activities. The field studies did not affect endangered or protected species.

### Taxon sampling

We obtained samples from eight accessions of *Avicennia*, including *A*. *germinans*, *A*. *officinalis*, *A*. *integra*, *A*. *alba*, *A*. *rumphiana*, and three varieties of *A*. *marina* (*A*. *marina* var. *marina*, *A*. *marina* var. *eucalyptifolia*, and *A*. *marina* var. *australasica*). The sample of *A*. *germinans* was collected from La Paz, Mexico. The detailed sampling information is listed in Table 1. To infer the phylogeny of *Avicennia* from all available data, we retrieved four sequences from two taxa, *A*. *bicolor* and *A*. *schaueriana* from GenBank (Table 1)[7]. We included *Thunbergia grandiflora* as the outgroup, since a close relationship between Thunbergioideae and *Avicennia* has been suggested based on recent molecular and morphological data [3, 12, 13]. Leaf tissue for DNA extraction was stored and dried in plastic bags with silica gel. Voucher specimens were deposited in the Herbarium of Sun Yat-Sen University (SYS).

**Table 1:** Distribution of *Avicennia* and voucher specimens.

Taxon	Locality	Voucher/Source
*A*. *alba* Blume	Bangkhunsai, Phetchaburi, Thailand	S.Shi 200908-AabBK01
*A*. *bicolor* Standl.	Playa Panama, Costa Rica	Nettle and Dodd, 2008
*A*. *germinans* (L.) Stearn	La Paz, Mexico	B. Liao 200901-AgeLP01
*A*. *integra* N. C. Duke	South Alligator River, North Territory, Australia	N.C.Duke 201412-AinDW01
		N.C.Duke 201412-AinDW02
*A*. *marina* (Forssk.) Vierh.		
var. *australasica*	Northcote, Auckland, New Zealand	M.Zhang 201106-AmaNZ01
var. *eucalyptifolia*	Cairns, Queensland, Australia	M.Zhang 201108-AmaCA01
var. *marina*	Wenchang, Hainan, China	S.Shi 200908-AmaWC01
	Kinabalu, Sabah, Malaysia	S.Shi 201108-AmaSB03
	Mida Creek, Kenya	Y.Deng 201104-AmaKY06
*A*. *rumphiana* Hallier f.	Kukup Johor, Malaysia	S.Shi 201108-AruKK01
*A*. *schaueriana* Stapf & Leechm. ex Moldenke	Macao, Brazil	Nettle and Dodd, 2008
*A*. *officinalis* L.	Laun, Ranong, Thailand	S.Shi 200908-AofLU01
	Sunderbans, Bangladesh	S.Shi 201102-AofSUN02
	Cilacap, Central Java, Indonesia	S.Shi 201210-AofCL01
*Thunbergia grandiflora* (Rottl. ex Willd.) Roxb.	Sun Yat-Sen University, Guangzhou, China	S.Shi 201306-GZ01

Voucher specimens are in the Herbarium of Sun Yat-Sen University (SYSU).

### DNA extraction, amplification and sequencing

The total genomic DNA of each individual was extracted by the modified CTAB method [14]. We used the *trnT-trnD* intergenic spacer region and the *psbA* gene for the phylogenetic reconstruction of all species of *Avicennia* because they are the only available sequence data from the two AEP species. *Avicennia bicolor* and *A*. *schaueriana* were not sampled in this study. For the detailed phylogenetic analyses of the IWP species, 25 pairs of primers were designed to amplify targeted nuclear genes from genomic DNA, based on the EST library of *A*. *marina* obtained from GenBank. The forward and reverse primers of each pair were located in different exons of the same gene to amplify a segment with introns. For most genes, only one band was amplified from all species. Two primer pairs produced PCR products with two bands, widely spaced in size. We re-designed allele-specific primers for these genes based on the sequences of isolated single bands that corresponded to the brightest-staining product that corresponded to the size of products from other taxa. The gene IDs, sequence lengths, gene descriptions, and primer sequences for the DNA markers are listed in S1 Table.

PCR was conducted with the following conditions: 94°C for 4 min, followed by 30 cycles of 94°C for 30 seconds, 48–58°C for 30 seconds, 72°C for 2 minutes, and a final extension of 8 minutes at 72°C. PCR products were purified using an agarose gel purification kit (QIAGEN, Hilden, Germany). Purified PCR products were subjected to direct sequencing. Sequencing reactions were conducted with amplification primers in an ABI 3730 DNA automated sequencer with BigDye chemistry (Applied Biosystems, Foster City, CA, USA). All sequences were deposited in GenBank. The accession numbers are listed in S2 Table.

### Phylogenetic analyses

Raw sequences were aligned and manually edited using SeqMan 7.1 (Madison, WI, USA). Multiple-sequence alignments were performed using ClustalW 2.1 [15] by leaving the default parameters unchanged and visually adjusting as necessary. Each gene alignment was tested with a 5% level chi-squared test for stationary base composition at variable sites using TREE-PUZZLE-5.2 [16].

We first reconstructed the phylogeny of *Avicennia*, with *Thunbergia grandiflora* as the outgroup, using the combined sequence data from two chloroplast segments. Prior to combining the sequences, congruence was examined using the partition–homogeneity test by PAUP* 4b10 [17, 18]. We further concatenated the sequences of 25 nuclear genes to infer the phylogenetic relationship among the IWP species, with *Avicennia germinans* as the outgroup.

Phylogenetic trees were reconstructed using Bayesian inference (BI), maximum likelihood (ML) and maximum parsimony (MP) methods. Prior to using model-based analytical approaches (BI and ML), nucleotide substitution models were selected based on the Akaike information criterion, as determined by jModelTest 2.1.4 [19]. For the chloroplast matrix, the best-fit model was a general time reversible model with a gamma correction for rate variation among sites (GTR + G). The best model for the nuclear gene matrix was the Hasegawa-Kishino-Yano model, with a gamma correction for rate variation among sites (HKY+G). Bayesian inference was performed using MrBayes 3.2.6 [20] with the models mentioned above. We ran the Markov chains for 10,000,000 generations, with one cold and three heated chains, starting from random trees and saving one out of every 1,000 samples. The runs were repeated twice. The resulting log-likelihood and number of generations were plotted to determine the point after which the log-likelihoods had stabilized. The remaining trees were used to calculate the posterior probabilities (P) through the construction of a majority rule consensus tree. Internodes with P > 95% were considered to be statistically significant. Maximum likelihood (ML) estimation was conducted with PHYML [21] utilizing the HKY+G best-fit model. Bootstrapping of the datasets was performed with 500 replications, and all node values for the ML trees are represented as the proportion of replicates in which that clade was recovered.

Maximum parsimony (MP) analyses were conducted using PAUP* 4.0b10 [18] with a “heuristic search”: tree bisection–reconnection branch swapping, MULPARS option on, and 1,000 random taxon additions. All characteristics were weighted equally, and gaps were treated as missing data. Subsequently, bootstrap analyses were performed on 500 replicates [22] using the options described above.

To evaluate the phylogenetic tree and to capture the major clusters of *Avicennia* species [23], a multi-dimensional scaling (MDS) analysis based on the matrix of the average pairwise genetic divergence value among all *Avicennia* species calculated using our sample of 2 chloroplast genes was conducted using the command "cmdscale" in R package *stats* [24]. The same analysis was conducted among IWP species calculated using our sample of 25 nuclear genes as well.

### Estimation of divergence time

The concatenated sequences of 25 nuclear genes were used to estimate the divergence times within *Avicennia*, with gaps treated as missing data. A Bayesian dating method with a relaxed molecular clock was implemented using the *mcmctree* program in PAML 4.8a [25]. *mcmctree* employs two strategies to model the change in evolutionary rate among lineages: independent rates and auto-correlated rates [26]. The HKY85+G model was used with different transition/transversion rate ratios (kappa) and different shape parameters (alpha) among the loci. A gamma prior G (2.6, 2) was assigned for kappa and G (1, 1) was assigned for alpha. The calibration point at the basal position of *Avicennia* was set as B (0.1, 0.5), with 100 million years as one time unit. The assumed range of divergence time (from 10 to 50 million years ago, MYA) between the IWP and AEP lineages was estimated by a previous study based on fossil records and molecular dating [27].

The overall substitution rate (rgene_gamma) was assigned by a gamma distribution with prior G(14, 40), which is a mean of 0.35×10^−8^ substitutions per site per year, based on the substitution rate from nuclear genes in genus *Avicennia* (He et al., in review). To estimate substitution rates per year for dating purpose, we constructed a phylogenetic tree using 2,326 orthologous genes from the following species: *Sesamum indicum*, *Avicennia officinalis*, *Avicennia marina*, *Acanthus leucostachyus* and *Acanthus ilicifolius* (S1 Fig). These have been sequenced in our lab or have published in genomes or transcriptomes [28, 29], as well as a fossil record [30]. We estimated species divergence time with fossil calibration using coding sequences (CDS). Since our data set included introns as well as CDS, we adjusted the substitutions rate (μ) by inferring the μ _intron_/μ_CDS_ using K_intron_/K_CDS_, where K is the number of segregating sites between *A*. *marina* and *A*. *alba* from 24 nuclear genes. We then calculated substitution rates for our sequences using weighted means of introns and CDS values. We saved 40,000 samples every two steps after discarding the initial 10,000 samples as the burn-in in *mcmctree*.

We next investigated the evolution of the flower characters among *Avicennia* species in IWP. We selected two morphologically important characters (stigma position and style length; S3 Table; [2, 31]) and reconstructed their ancestral states. The two characters were coded and scored following morphological descriptions in the literature [1, 2, 6, 32] and our field observations. The data matrixes used for the analyses are presented in the S4 Table. Based on the phylogenetic tree reconstructed with 25 nuclear genes, we performed our analyses using maximum parsimony in Mesquite v. 3.04 [33].

## Results

### The phylogeny of *Avicennia* based on combined chloroplast sequences

**Fig 1:**
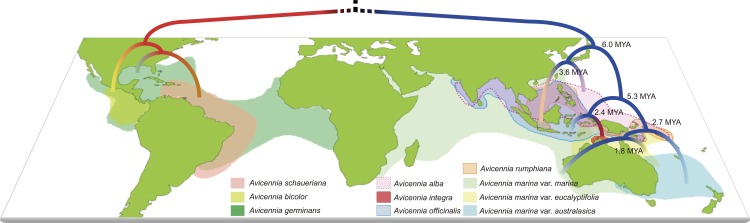
Distribution and the phylogenetics of *Avicennia*. Distributions of species are color coded on the map (modified from [34]). The map was modified from the 1:110m coastline map of Natural Earth (http://www.naturalearthdata.com). The phylogenetic relationship between species is based on the phylogenic analyses from chloroplast and nuclear genes. The divergence time for species in the Indo-Western Pacific (IWP) region was calibrated by *mcmctree 4*.*8a* [25].

All gene segments we obtained satisfied the stationarity test (P > 5%) for base composition at the variable sites and were retained for further analysis. The partition–homogeneity test indicated that the *trnT-trnD* and *psbA* sequences could be combined for phylogenetic analyses (P = 1.0). Phylogenetic analyses of this dataset using the BI, ML and MP methods yielded identical topologies (Fig 1; see detail in S2 Fig).

In the combined chloroplast tree, species of *Avicennia* fell into two clades: five in the IWP region forming one clade and the remaining three in the AEP region forming the other (S2 and S3 Figs; Posterior Probability P = 1.00 for BI; Bootstraps BS = 100% and 99% for ML and MP, respectively). Long branches between the crown node and the roots of both the IWP and AEP clades indicated that the two clades had evolved independently over a long period since the initial split from the common ancestor. Within the AEP lineage, *A*. *germinans* was the sister to *A*. *schaueriana* (P = 0.99; BS = 78% and 72% for ML and MP, respectively). Within the IWP clade, *A*. *officinalis* was the sister to *A*. *integra*, with a high level of support (P = 1.00 and BS = 88% and 95%). Those two species were close to the three varieties of the *A*. *marina* complex (P = 0.99; BS = 70% and 65%). The phylogenetic positions of *A*. *alba* and *A*. *rumphiana* were still unclear in the chloroplast tree (P < 0.5 and BS < 50%).

### Phylogenetic relationships among the IWP species based on 25 nuclear genes

**Fig 2:**
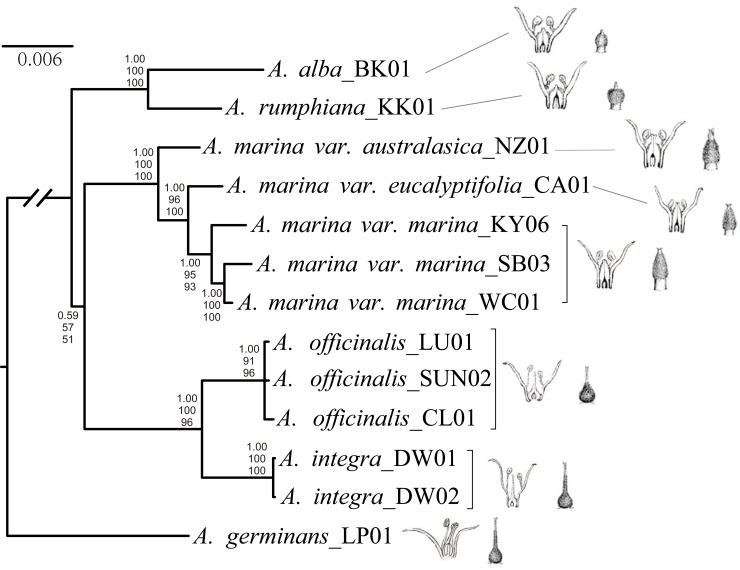
Bayesian Inference tree of *Avicennia* species in the Indo-West Pacific (IWP) region. Based on concatenated sequences of 25 nuclear genes, with A. *germinans* as the outgroup. Bayesian posterior probabilities for Bayesian Inference (BI), Likelihood bootstrap values from the maximum likelihood analysis (ML) and Parsimony bootstrap values from maximum parsimony analysis (MP) are indicated at nodes (BI/ML/MP). Two selected morphological characters of taxonomic importance (source from [1]) have been mapped on the tree.

**Fig 3:**
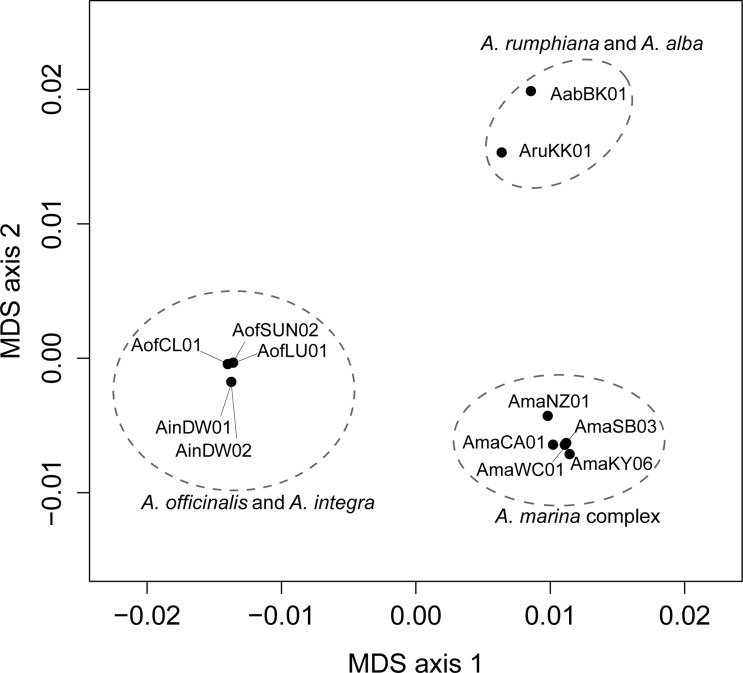
Multidimensional scaling plot. Plot of the first and second axis of a multidimensional scaling matrix based on pairwise genetic divergence value among IWP species of 25 nuclear genes. The numbers and letters refer to sample identity is listed in Table 1.

We conducted further phylogenetic analyses among the IWP species using concatenated sequences of 25 nuclear genes, with *Avicennia germinans* as an outgroup. The BI, ML, and MP methods yielded identical topologies (only the BI tree is shown in Fig 2). The results suggest that the IWP clade could be further split into three distinct subclades (Fig 2). *Avicennia alba* and *A*. *rumphiana* were grouped into one subclade (P = 1.00 and BS = 100% for both ML and MP). The three varieties of *A*. *marina* formed another subclade (P = 1.00, BS = 100% for ML and MP). Among the three varieties, *A*. *marina*, var. *marina* is closer to *A*. *marina* var. *eucalyptifolia* than to *A*. *marina* var. *australasica* (P = 1.00, BS = 96% for ML and BS = 100% MP). *Avicennia integra* and *A*. *officinalis* were grouped into the third subclade (P = 1.00, BS = 100% for ML and BS = 96% for MP), which was consistent with results based on the chloroplast segments (S2 Fig). We had low power to resolve the sub-clade relationships, but our results suggest that the *A*. *officinalis*/*A*. *integra* subclade is more closely related to the *A*. *marina* complex than to the *A*. *alba/A*. *rumphiana* subclade (P = 0.59; BS = 57% and 51% for ML and MP, respectively). If we concatenate the chloroplast and nuclear genes and repeat phylogenetic reconstruction using three methods as before, the clustering pattern is the same, with increased support rate for this node (P = 0.72; BS = 69% and 58% for ML and MP, respectively; P = 1.00 for partition–homogeneity test. See the S4 Fig) MDS analysis of the mean pairwise genetic divergence among IWP species, again based on our sample of 25 nuclear genes, revealed a consistent pattern, with *A*. *officinalis* and *A*. *integra*, *A*. *alba* and *A*. *rumphiana*, and the three varieties of *A*. *marina* forming clear groups identical to those revealed by our phylogenetic analyses (Fig 3).

Fig 1 shows divergence time estimates. Within the IWP clade, the *A*. *alba*/*A*. *rumphiana* subclades first diverged *c*. 6.0 MYA, while the other two subclades diverged from each other *c*. 5.3 MYA. Within the subclades, the initial split between *A*. *alba* and *A*. *rumphiana* was estimated to be *c*. 3.6 MYA, whereas the split between *A*. *officinalis* and *A*. *integra* was estimated to have occurred *c*. 2.4 MYA. The two divergence events within the *A*. *marina* complex were estimated to have occurred at 2.7 and 1.8 MYA, respectively.

Molecular phylogenies can shed light on evolution of morphological traits. Therefore, we selected two important flower structure phenotypes: stigma position and style length. Ancestral states reconstruction for stigma position is shown in the S5 Fig, while the results for style length are in the S6 Fig. Our analyses indicate that the ancestral stigma position in *Avicennia* from IWP is between anthers, and the ancestral style length is over 1.0 mm. These results suggest that *A*. *officinalis* retains ancestral state in flower structure, while *A*. *alba*/*A*. *rumphiana* lineage and the *A*. *marina* complex exhibit independent degeneration.

## Discussion

### Phylogenetic relationships within *Avicennia*

The classification and phylogeny of *Avicennia* were first studied by Tomlinson (1986) [2], Duke (1990, 1991) [1, 6] and Duke et al., (1998) [5] based on morphological and allozyme data. Some studies on phylogeographic patterns of this genus based on a few nuclear and chloroplast DNA markers have been conducted for three AEP species of *Avicennia* [8, 9]. The present study is the first to provide a phylogenetic reconstruction of *Avicennia* based on multiple loci and comprehensive sampling (Fig 1 & S2 and S3 Figs). Our phylogeny reveals with high confidence that *Avicennia* is divided into two distinct clades, consistent with our expectations based on geography and systematics.

Using nuclear gene sequences sampled in this study, we were able to resolve phylogenetic relationships among five species of the IWP region. Our analyses suggest three major subclades within the IWP clade: (1) *A*. *rumphiana* and *A*. *alba*, (2) *A*. *officinalis* and *A*. *integra*, and (3) the *A*. *marina* complex (Figs 2 and 3). Interestingly, these subclades can be clearly differentiated using two morphological traits in floral structure, i.e. stigma position in relation to the anthers, and the length of styles (Fig 2). In previous studies, species of *Avicennia* in the IWP region were divided into two lineages, the Officinalis lineage and the Marina lineage, based on the main types of corolla [2]. The Officinalis lineage includes *A*. *officinalis* and *A*. *integra*, while the latter includes all other species. Our molecular phylogenetic analyses strongly support a subclade with *A*. *officinalis* and *A*. *integra* (Fig 2). In fact, those two species share some taxonomically important morphological characters (e.g., stamens markedly unequal; one pair is short and the one pair is long). Both species have an elongate flask- or ampulla-shape style exerted above or equal to the upper edge of anthers. The length of style is about 2mm (Fig 2, [1]).

The *A*. *marina* complex is the sister to the *A*. *officinalis*/*A*. *integra* subclade and is not grouped with the other two species, *A*. *rumphiana* and *A*. *alba*, which were included in the *Marina* lineage by Tomlinson (1986) [2]. Structural attributes of the floral organs of *A*. *rumphiana* and *A*. *alba* differ from those of the *A*. *marina* complex. The style of *A*. *rumphiana* and *A*. *alba* is minute and narrow, ending well below the anthers. The length of style is from 0.2–0.4mm [1]. In contrast, the stigma is positioned level with the middle or the lower edge of the anthers in the *A*. *marina* complex. The style is about 0.5mm [1].

Our phylogenetic tree suggests that *A*. *rumphiana* and *A*. *alba* are closely related to each other and form a basal subclade in the IWP lineage. Previous morphological analyses produced conflicting results regarding their affinity to other species of *Avicennia*. For example, an analysis of flower, fruit and leaf data [6] showed *A*. *alba* to be closely related to the *A*. *marina* complex, but positioned *A*. *rumphiana* as an independent group [32, 35]. However, an analysis of 22 morphological attribute means, such as width and length of the leaves, flowers, and fruit, as well as the inflorescence, stigma position, and propagule shape, suggested that *A*. *rumphiana* and *A*. *alba* were most closely related to each other [6], which is supported by the present study.

We used our molecular phylogeny to study evolution of flower morphology in *Avicennia*. We infer the ancestral of two important traits: stigma position and style length (S5 and S6 Figs). Our results indicated the ancestral flower structure is preserved in the *A*. *officinalis*/*A*. *integra* lineage, with style length about 2mm and stigma level in the middle of the variety anthers (Fig 2). The style degenerated and the stigma became positioned under the anthers in the other IWP *Avicennia* species, especially for *A*. *abla* and *A*. *rumphiana*. Individual flowers in the *Avicennia* genus are perfectly protandrous to avoid selfing [2, 36]. The change of relative positions of anthers and stigma indicates that these species might have evolved herkogamy as a supplemental mechanism for selfing avoidance [37, 38]. Li et al., (2013) [37] suggest that herkogamy is more effective than dichogamy for avoiding reduction of fitness through selfing. Additional field observations and investigations of flower phenology in *Avicennia* species are needed to establish evolutionary significance of this observation.

### Diversification and Evolution within *Avicennia* in IWP

Although the first fossil record of *Avicennia* in the IWP region dates back to the late Eocene of southwest Australia, significant flourishing of *Avicennia* in this region has occurred since the Miocene [39–41]. The distribution of ancestral *Avicennia* was likely to have been similar to its present location, extending from Japan to Borneo and from the Marshall Islands to the Red Sea [40, 41]. In this study, diversification of the species of *Avicennia* in the IWP region was dated at approximately 6.0 MYA (95% HPD: 2.8~9.7 MYA; Fig 2). The distribution of the major biogeographic barriers and the shallow seas in the IWP region has not changed significantly since that time [42]. Sea levels, however, began to fluctuate because of climate change [43]. Lowered sea levels resulted in the emergence of land barriers in the IWP region [44], particularly within the Indo-Australia archipelago, which may have facilitated isolation of the *Avicennia* populations and the subsequent diversification of this clade throughout the region. A number of studies of marine species have also suggested a diversification surrounding the IWP oceans in that period (late Miocene-Pliocene), such as wrasse (*Halichoeres* in 3.5–8 MYA [45]), *Pomacentrus coelestis* species complex (within 2.8 MYA [46]) and mangrove snails (*Cerithidea*, within Plio-Pleistocene [47]).

As the most dominant *Avicennia* species in this area, *A*. *marina* displayed a similar evolutionary history based on the present study. In our phylogenetic tree, the crown age of the *A*. *marina* subclades was dated at 2.8 MYA (95% HPD: 1.2~4.6 MYA; Fig 2), which is consistent with the age estimated for this subclade based on allozyme data (approximately 2 MYA, [5]). These estimates support the conclusion that varieties of *A*. *marina* diverged more recently, in the Pleistocene, possibly during periods of lowered sea levels during periods of glaciation [43]. Based on genetic diversity of three *A*. *marina* varieties, Duke et al., (1998) [5] suggested that the split occurred during the ice age, when the emerged land barrier between New Guinea and Australia isolated the populations on the west coast (var. *marina*) from those on the east (var. *eucalyptifolia*). Population genetic data have revealed the role of land barriers in shaping genetic structure across IWP in many species, such as *Kandelia candel* [48, 49], *Ceriops spps*., [50–52], *Bruguiera gymnorhiza* [53], *Rhizophora apiculata* [54], reef fish [55] and starfish [56]. However, the divergence between var. *australasica* and var. *eucalyptifolia* along the coast of East Australia is difficult to explain without positing an additional barrier in this region. This barrier has not been identified in these prior studies [5]. Hewitt (2000) [57] proposed that sea level oscillations would lead to many changes in distribution of marine and coastal species in IWP. Considering repeated oscillations of sea level, *Avicennia marina* populations, especially var. *eucalyptifolia* near the Strait of Torres, may experienced several rounds of isolation, dispersal and admixture. Wide sampling and population-genomic studies are necessary to reveal the demography of *Avicennia marina* and to understand the causes of evolution and diversification of *Avicennia* species.

## Conclusions

*Avicennia* consists of two monophyletic clades: Atlantic-East Pacific (AEP) lineage and Indo-West Pacific (IWP) lineage. Three distinct subclades were identified within the IWP group with high bootstrap support: (1) *A*. *rumphiana* and *A*. *alba*, (2) *A*. *officinalis* and *A*. *integra*, and (3) the *A*. *marina* complex. The result was also supported by two morphological traits in floral structure: stigma position in relation to the anthers and the length of the styles. The diversification of the IWP lineage was dated to late Miocene (*c*. 6 MYA) and may have been driven largely by fluctuating sea levels since that time.

## Supporting Information

S1 Fig The divergence time of *Avicennia* group. Bars show the error bar of 95% confidential interval of divergence time. Red rectangle shows the earliest fossil records of mangrove lineages while gray one shows that of inland relatives. The time range of fossils are the earliest and most conformed fossil was from Spain and dated to Middle Bartonia (38.3–39.4 MYA, [30]).
(TIF)

S2 Fig Bayesian inference tree of *Avicennia* inferred from combined chloroplast sequences. Bayesian posterior probabilities for Bayesian Inference (BI), Likelihood bootstrap values for maximum likelihood (ML) and Parsimony bootstrap values for maximum parsimony (MP) are indicated at nodes (BI/ML/MP), respectively. IWP: Indo-West Pacific region; AEP: Atlantic-East Pacific region.
(TIF)

S3 Fig Multidimensional scaling plot. Plot of the first and second axis of a multidimensional scaling matrix based on pairwise genetic divergence value among all *Avicennia* species of two chloroplast genes.
(TIF)

S4 Fig Bayesian Inference tree of *Avicennia* species in the Indo-West Pacific (IWP) region from combined chloroplast genes and nuclear genes. Based on concatenated sequences of chloroplast gene and 25 nuclear genes, with A. *germinans* as the outgroup. Bayesian posterior probabilities for Bayesian Inference (BI), Likelihood bootstrap values from the maximum likelihood analysis (ML) and Parsimony bootstrap values from maximum parsimony analysis (MP) are indicated at nodes (BI/ML/MP).
(TIF)

S5 Fig Inference of evolution of stigma position for Avicennia species in IWP based on phylogenetic tree in Fig 2. The coding and its meaning were showed in legend.
(TIF)

S6 Fig Inference of evolution of style length for Avicennia species in IWP based on phylogenetic tree in Fig 2. The coding and its meaning were showed in legend.
(TIF)

S1 Table Data from molecular markers. (DOCX)

S2 Table GenBank accessions. (DOCX)

S3 Table The coding and scoring for the characters in this study. (DOCX)

S4 Table The data matrix. (DOCX)
