# Supplementary figures and images for: Re-Evaluation of Phylogenetic Relationships among Species of the Mangrove Genus Avicennia from Indo-West Pacific Based on Multilocus Analyses
Source: PLoS One. 2016 Oct 7;11(10):e0164453. doi: 10.1371/journal.pone.0164453 (PMC5055292; doi:10.1371/journal.pone.0164453)

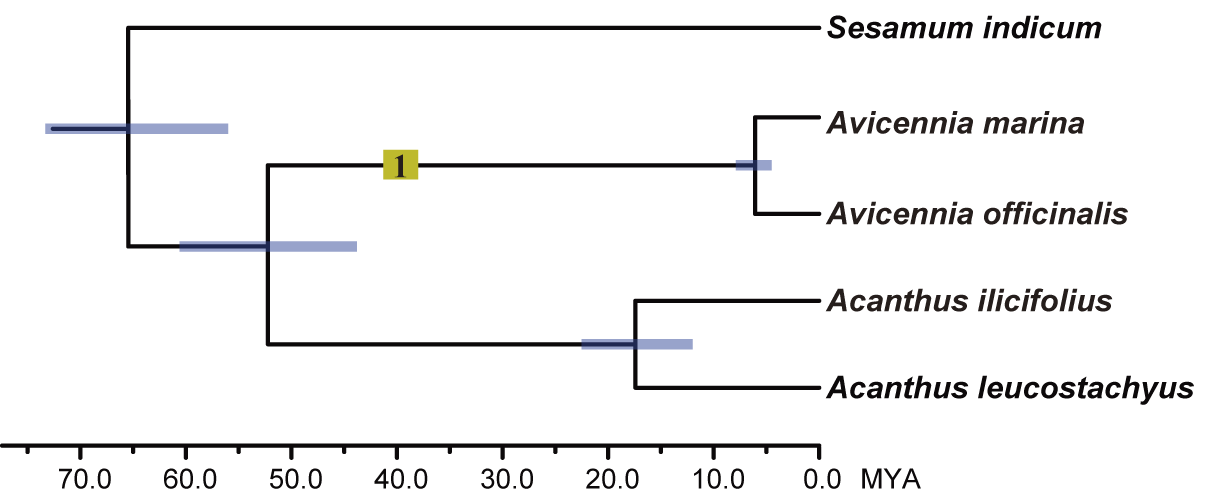

Supplement: S1 Fig — Bars show the error bar of 95% confidential interval of divergence time. Red rectangle shows the earliest fossil records of mangrove lineages while gray one shows that of inland relatives. The time range of fossils are the earliest and most conformed fossil was from Spain and dated to Middle Bartonia (38.3–39.4 MYA, [30]). (TIF) [file pone.0164453.s001.tif]

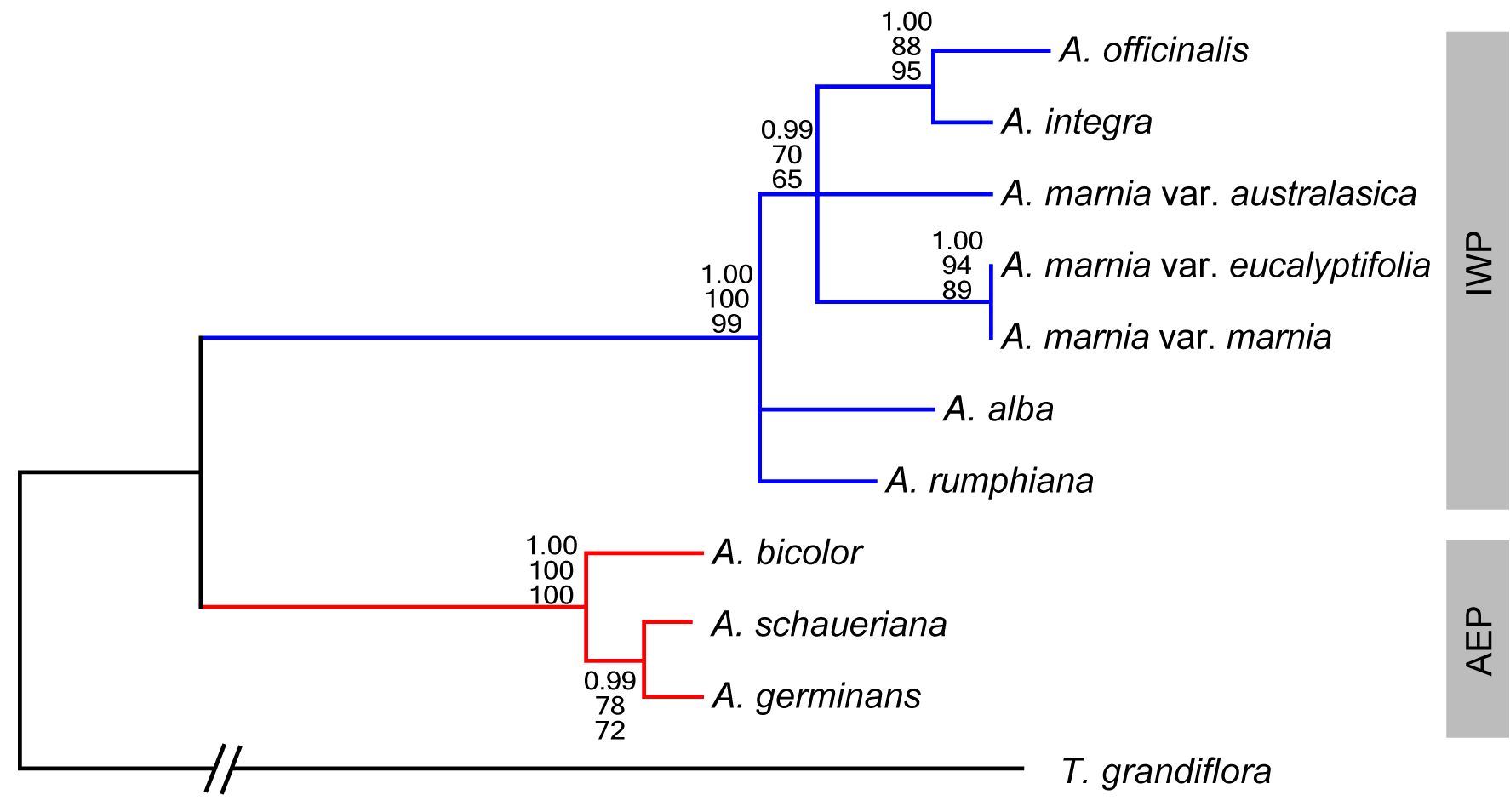

Supplement: S2 Fig — Bayesian posterior probabilities for Bayesian Inference (BI), Likelihood bootstrap values for maximum likelihood (ML) and Parsimony bootstrap values for maximum parsimony (MP) are indicated at nodes (BI/ML/MP), respectively. IWP: Indo-West Pacific region; AEP: Atlantic-East Pacific region. (TIF) [file pone.0164453.s002.tif]

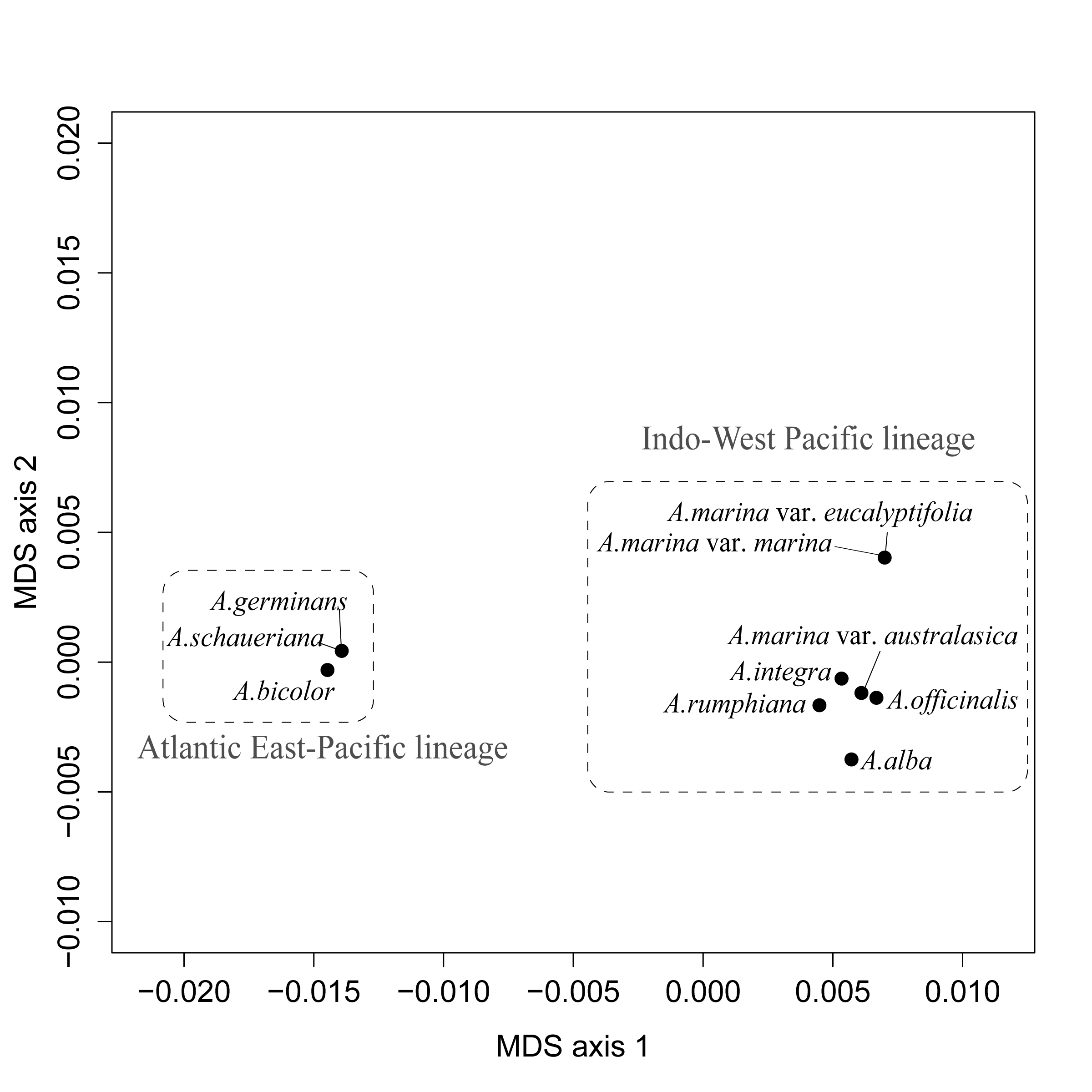

Supplement: S3 Fig — Plot of the first and second axis of a multidimensional scaling matrix based on pairwise genetic divergence value among all Avicennia species of two chloroplast genes. (TIF) [file pone.0164453.s003.tif]

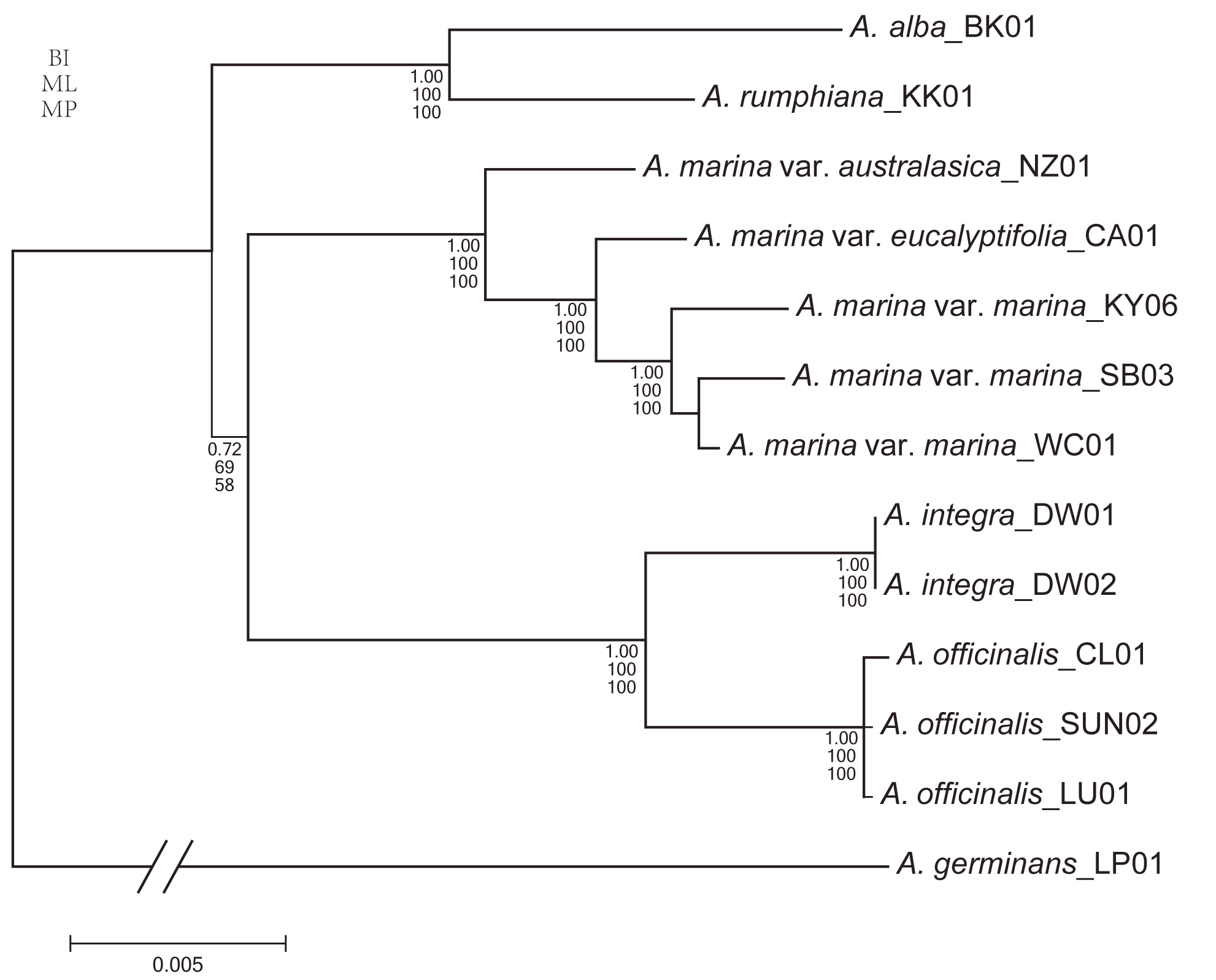

Supplement: S4 Fig — Based on concatenated sequences of chloroplast gene and 25 nuclear genes, with A. germinans as the outgroup. Bayesian posterior probabilities for Bayesian Inference (BI), Likelihood bootstrap values from the maximum likelihood analysis (ML) and Parsimony bootstrap values from maximum parsimony analysis (MP) are indicated at nodes (BI/ML/MP). (TIF) [file pone.0164453.s004.tif]

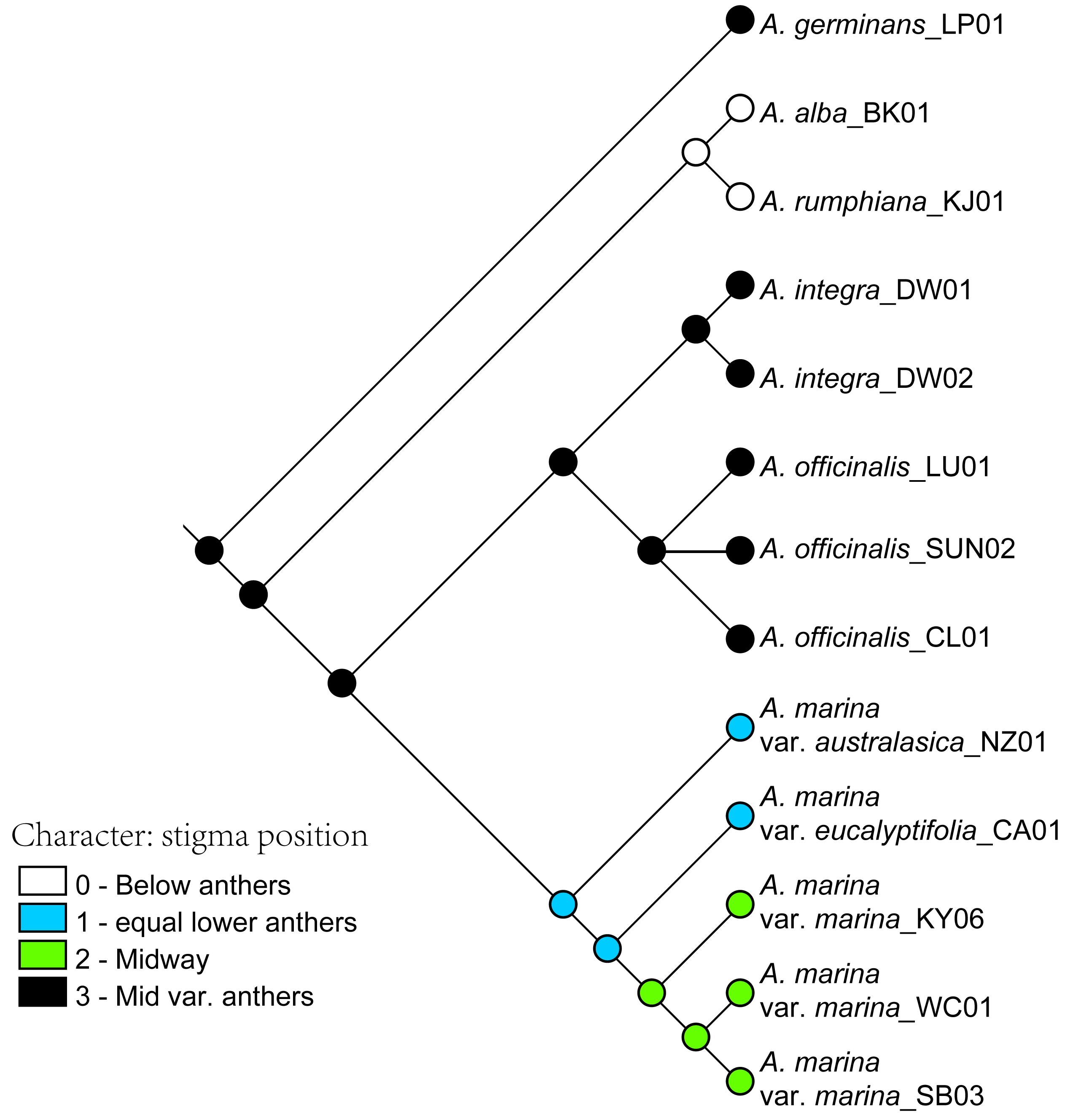

Supplement: S5 Fig — The coding and its meaning were showed in legend. (TIF) [file pone.0164453.s005.tif]

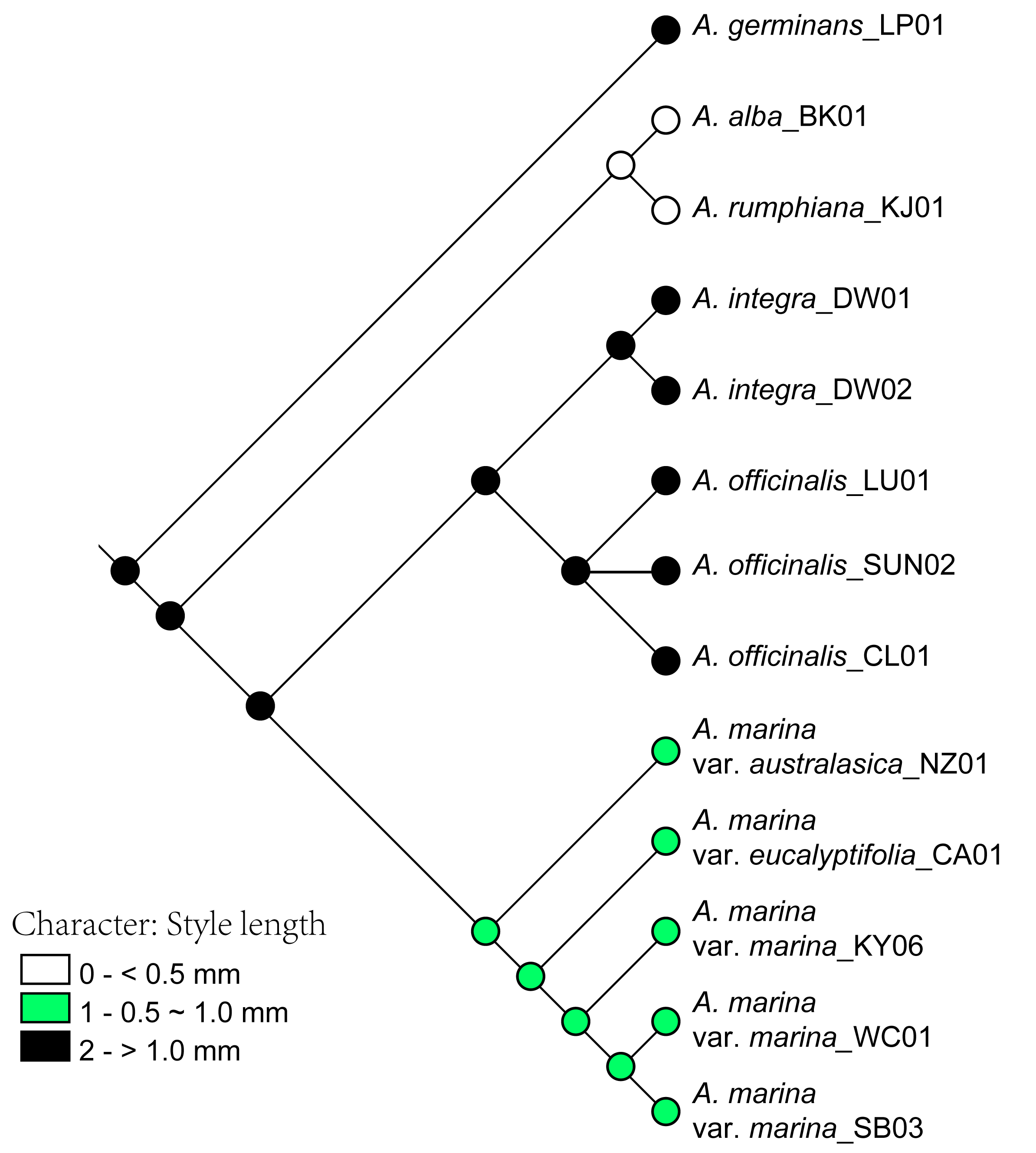

Supplement: S6 Fig — The coding and its meaning were showed in legend. (TIF) [file pone.0164453.s006.tif]
