# Supplementary material for: Re-Evaluation of Phylogenetic Relationships among Species of the Mangrove Genus Avicennia from Indo-West Pacific Based on Multilocus Analyses
Source: PLoS One. 2016 Oct 7;11(10):e0164453. doi: 10.1371/journal.pone.0164453 (PMC5055292; doi:10.1371/journal.pone.0164453)
Supplement: S1 Table — (DOCX) [file pone.0164453.s007.docx]

**S1 Table. Information of molecular markers**

| **Gene ID** | **Primers Sequence (5’-3’)** | **Gene Annotation** | **Annealing temperature (ºC)** | **Effective length (bp)** |
| --- | --- | --- | --- | --- |
| trnD-trnT | F:ACCAATTGAACTACAATCCC  R:CCCTTTTAACTCAGTGGTAG | chloroplast intergenic spacer | 55 | 697 |
|  |  |  |  |  |
| psbA | F:GTWATGCAYGAACGTAATGCTC R:CGCGCATGGTGGATTCACAATCC | chloroplast gene | 53 | 401 |
|  |  |  |  |  |
| 0009a | F:AGGATATTCTGAAGCAGCCAAAGC R:TCTTGAAGGAGGTGCAGGGAGA | Phosphoinositide phospholipase C 2 | 60.5 | 535 |
| 0056a | F:GTCCACAGAAACCCAGACATCC R:TAGAACAAGGAGAACTAGACTTTTACTCAG | E3 ubiquitin-protein ligase MARCH6 | 59 | 790 |
| 0101 | F:CAACTACTTCTTCCTCAATGCCACA R:GCTTATAGAATCAGTTCAGACCCATACA | Cathepsin B | 60 | 836 |
| 0154 | F:TGCACGGGCAGCTTCAGCA R:GGTGGAACTAGCAAGTGGAACATCA | Polyadenylate-binding protein, cytoplasmic and nuclear | 58.5 | 590 |
| 0226 | F:CGTTTTCTGCTTCATCTACCGTCAT R:CAGGGATTCCGACAGGTTGTTG | Nudix hydrolase 18, mitochondrial | 54 | 742 |
| 0229 | F:GAGATAAGCACCCAACGCATTACTC R:GGAAGGTCACCAACCCCACG | Probable polyketide synthase 13 | 58.4 | 488 |
| 0256 | F:CCACTGCACCTTTATGCCCTTGA R:CGTCCGGTTGTCGATTTGTCCT | Serine-threonine kinase receptor-associated protein | 63.8 | 1493 |
| 0257 | F:CTGACACTGCACATCAATCCCACT R:TCCTCTTTATTCTGTTTATGCCGCTAC | Estradiol 17-beta-dehydrogenase 12 | 61.4 | 653 |
| 0258 | F:AACAGGGCAGGCTTCTTGG R:AGGGCTGATGCTGACGCTT | NADH dehydrogenase [ubiquinone] iron-sulfur protein 8-B, mitochondrial | 62.3 | 813 |
| 0259 | F:TCTCGCCAGGAAACAGAGGC R:CTTTGTCGTATGTCCATCGTGGTA | Shaggy-related protein kinase alpha | 62.3 | 797 |
| 0279 | F:CAACAGAGCTTCAGCGGTCT R:GCTTTTCATCTTCACAGCACAA | Unknown protein | 57 | 463 |
| 0291 | F:GAAGCAACAGCCAGTCATCACATC R:GGTGGTAGCGGGCTTGGTCT | Ethylene receptor 1 | 60 | 978 |
| 0347 | F:CACGCTTCCAATCTTTAGATCACCCT R:GGAGACACCACAGGCACCAACC | Abscisic acid receptor PYL9 | 60.6 | 634 |
| 0349a | F:GAGCTCCACCTTCTTCTTGGTCTTGTA R:ACGGGCCGTTAGCCATTTCG | Heavy metal-associated isoprenylated plant protein 26 | 56.6 | 629 |
| c019 | F:GCTTTCACCGCAGCAGGACA R:TCACTCGCAGGGAATTATTCATCG | Serine/threonine-protein kinase HT1 | 57.2 | 578 |
| c038 | F:CGGGTTCTTCAACAGCTTTGC R:TTGGAAGGTCAGGATGAAAGTG | Vacuolar protein sorting-associated protein 35B | 54.7 | 827 |
| c099 | F:GCAGCAAGTCCTCCATAGACAACCT R:GATACTGGTCCACCTGCCACAAA | Heat shock 70 kDa protein 14 | 56 | 568 |
| c119 | F:GGCACCCCACGAAGATAAAGA R:ACCGTACCAATCAACCCAAAGC | Probable small nuclear ribonucleoprotein F | 51 | 844 |
| c121 | F:GTGCTGAGTATCGACATCTTTATCC R:ATGAAGTTCCTCGGTTGCGTA | Alpha-glucan phosphorylase, H isozyme | 51 | 1076 |
| c129 | F:GGTAGCCCATGCGATTAA R:GGAGCAAAGATGTGGTGAA | 60S ribosomal protein L38 | 53.3 | 614 |
| c138 | F:TGGCACCAACAAGCCTGTCAAT R:TCCAGAAGGGCGTCGAGTCTAA | Thioredoxin H-type 1 | 51 | 998 |
| c202 | F:ATCCCATCCTTTCAATCATA R:GTTCGTCATCAGGTGGTG | Uncharacterized protein Rv1829/MT1877 | 54 | 731 |
| c221 | F:TCAGTGAAAACCAGAACG R:GGCAGCTACGCAAGAAAA | Translocase of chloroplast 34 | 49.8 | 577 |
| c244 | F:CGGATATTGACTGATCTC R:AGCCTCTCTCTTCGTTAG | Ubiquitin thioesterase otubain-like | 52.8 | 716 |
| c285 | F:AATGAAAGAGTTGGTGCTA R:CTGTGACAAAGGCAAGGG | Putative dihydrodipicolinate reductase 3, chloroplastic | 51.1 | 670 |
