# Supplementary material for: Re-Evaluation of Phylogenetic Relationships among Species of the Mangrove Genus Avicennia from Indo-West Pacific Based on Multilocus Analyses
Source: PLoS One. 2016 Oct 7;11(10):e0164453. doi: 10.1371/journal.pone.0164453 (PMC5055292; doi:10.1371/journal.pone.0164453)
Supplement: S2 Table — (DOCX) [file pone.0164453.s008.docx]

**S2 Table. GenBank accessions**

| Species | Individual | *psbA* | *trnD-trnT* | *0009a* | *0056a* | *101* | *154* | *226* | *229* | *256* |
| --- | --- | --- | --- | --- | --- | --- | --- | --- | --- | --- |
| *A. abla* | AabBK01 | KT453639 | KT453652 | KP025973 | KP026059 | KP025981 | KP025989 | -- | KT453675 | KP026013 |
| *A. rumphiana* | AruKK01 | KT453643 | KT453650 | KP025980 | KP026065 | KP025987 | KP025995 | KP026002 | KP026012 | KP026019 |
| *A. officinalis* | AofLU01 | KT453644 | KT453649 | KT453671 | KT453685 | KT453672 | KT453673 | KP026000 | KT453677 | KT453679 |
|  | AofSUN02 | -- | KX812719 | KX240376 | KX240402 | KX240432 | KX240459 | -- | KX240483 | -- |
|  | AofCL01 | -- | KX812715 | KX240377 | KX240404 | KX240434 | KX240461 | -- | KX240485 | -- |
| *A. integra* | AinDW01 | KT453637 | KT453646 | KT453655 | KT453656 | KT453657 | KT453658 | KT453659 | KT453660 | KT453661 |
|  | AinDW02 | -- | -- | KX240357 | KX240381 | KX240408 | KX240438 | -- | KX240465 | -- |
| *A. marina* var*. australasica* | AmaNZ01 | KT453638 | KT453651 | KP025977 | KP026063 | KP025985 | KP025993 | KP025999 | KP026008 | KP026017 |
| *A. marina* var*. eucalyptifolia* | AmaCA01 | KT453641 | KT453654 | KP025975 | KP026061 | KP025983 | KP025991 | KT453674 | KT453676 | KT453678 |
| *A. marina* var*. marina* | AmaWC01 | KT453642 | KT453648 | KP025976 | KP026062 | KP025984 | KP025992 | KP025998 | KP026007 | KP026016 |
|  | AmaKY06 | -- | KX812716 | KX259012 | KX259013 | KX259015 | KX259017 | -- | KX259019 | -- |
|  | AmaSB03 | -- | KX812717 | KX259011 | KX259014 | KX259016 | KX259018 | -- | KX259020 | -- |
| *A. germinans* | AgeLP01 | KT453640 | KT453653 | KP025974 | KP026060 | KP025982 | KP025990 | KP025996 | KP026004 | KP026014 |
|  |  |  |  |  |  |  |  |  |  |  |
| Species | Individual | *257* | *258* | *259* | *279* | *291* | *347* | *0349a* | *c019* | *c038* |
| *A. abla* | AabBK01 | KP026020 | KP026027 | KP026034 | KP026041 | KP026046 | KP026052 | KT453698 | KP026072 | KP026107 |
| *A. rumphiana* | AruKK01 | KP026026 | KP026033 | KP026040 | KP026045 | KP026051 | KP026058 | KT453699 | KP026078 | -- |
| *A. officinalis* | AofLU01 | -- | KT453681 | KT453682 | KP026044 | KT453683 | KP026057 | KT453703 | KP026077 | KP026112 |
|  | AofSUN02 | -- | KX240509 | -- | -- | -- | KX240534 | -- | KX240559 | -- |
|  | AofCL01 | -- | KX240511 | -- | -- | -- | KX240538 | -- | KX240561 | -- |
| *A. integra* | AinDW01 | -- | KT453662 | KT453663 | -- | KT453664 | -- | -- | -- | KT453665 |
|  | AinDW02 | -- | KX240489 | -- | -- | -- | KX240515 | -- | KX240542 | -- |
| *A. marina* var*. australasica* | AmaNZ01 | KP026025 | KP026031 | KP026038 | KP026043 | KP026049 | KP026056 | KT453701 | KP026076 | KP026110 |
| *A. marina* var*. eucalyptifolia* | AmaCA01 | KP026023 | KT453680 | KP026036 | -- | KP026047 | KT453684 | KT453700 | KT453689 | KP026109 |
| *A. marina* var*. marina* | AmaWC01 | KP026024 | KP026030 | KP026037 | -- | KP026048 | KP026055 | KT453702 | KP026075 | KT453696 |
|  | AmaKY06 | -- | KX259021 | -- | -- | -- | -- | -- | -- | -- |
|  | AmaSB03 | -- | KX259022 | -- | -- | -- | -- | -- | -- | -- |
| *A. germinans* | AgeLP01 | KP026021 | KP026028 | KP026035 | KP026042 | -- | KP026053 | -- | KP026073 | KP026108 |
|  |  |  |  |  |  |  |  |  |  |  |
| Species | Individual | *c099* | *c119* | *c121* | *c129* | *c138* | *c202* | *c221* | *c244* | *c285* |
| *A. abla* | AabBK01 | KP026113 | KT453704 | KT453713 | KT453720 | KT453686 | KP026079 | KT453691 | -- | KT453694 |
| *A. rumphiana* | AruKK01 | KP026119 | KT453706 | KT453712 | KT453721 | KP026071 | KP026084 | KP026092 | KP026098 | KP026106 |
| *A. officinalis* | AofLU01 | KP026118 | KT453705 | KT453714 | KT453719 | KP026070 | KP026083 | KP026091 | KT453693 | KP026105 |
|  | AofSUN02 | KX240584 | -- | -- | -- | KX240613 | KX240639 | -- | KX240663 | KX240687 |
|  | AofCL01 | KX240586 | -- | -- | -- | KX240614 | KX240640 | -- | KX240665 | KX240690 |
| *A. integra* | AinDW01 | -- | -- | -- | -- | KT453666 | KT453667 | KT453668 | KT453669 | KT453670 |
|  | AinDW02 | KX240564 | -- | -- | -- | KX240590 | KX240618 | -- | KX240644 | KX240669 |
| *A. marina* var*. australasica* | AmaNZ01 | KP026116 | KT453709 | KT453711 | KT453717 | -- | KP026082 | KP026090 | KP026096 | KP026104 |
| *A. marina* var*. eucalyptifolia* | AmaCA01 | KP026115 | KT453707 | -- | KT453718 | KT453687 | KT453690 | KT453692 | KP026094 | KT453695 |
| *A. marina* var*. marina* | AmaWC01 | KT453697 | KT453708 | KT453710 | KT453716 | KT453688 | -- | KP026089 | KP026095 | KP026103 |
|  | AmaKY06 | -- | -- | -- | -- | -- | -- | -- | -- | KX259023 |
|  | AmaSB03 | -- | -- | -- | -- | -- | -- | -- | -- | KX259024 |
| *A. germinans* | AgeLP01 | KP026114 | -- | KT453715 | -- | KP026067 | KP026080 | KP026086 | KP026093 | KP026100 |
