# Supplementary material for: Re-Evaluation of Phylogenetic Relationships among Species of the Mangrove Genus Avicennia from Indo-West Pacific Based on Multilocus Analyses
Source: PLoS One. 2016 Oct 7;11(10):e0164453. doi: 10.1371/journal.pone.0164453 (PMC5055292; doi:10.1371/journal.pone.0164453)
Supplement: S3 Table — (DOCX) [file pone.0164453.s009.docx]

S3 Table. The coding and scoring for the characters in this study.

| Characters | Character states |
| --- | --- |
| 1. Stigma position | 0 - Below anthers  1 - equal lower anthers  2 - midway  3 - Mid var. anthers^a^ |
| 2. Style length | 0 - <0.5 mm  1 - 0.5 ~ 1 mm  2 - >1 mm |

a) That means the stigma erected excess over the short pair of anther, but below the long pair.
