# Supplementary material for: Re-Evaluation of Phylogenetic Relationships among Species of the Mangrove Genus Avicennia from Indo-West Pacific Based on Multilocus Analyses
Source: PLoS One. 2016 Oct 7;11(10):e0164453. doi: 10.1371/journal.pone.0164453 (PMC5055292; doi:10.1371/journal.pone.0164453)
Supplement: S4 Table — (DOCX) [file pone.0164453.s010.docx]

S4 Table. The data matrix.

|  | Stigma position | Style length |
| --- | --- | --- |
| *A. officinalis* | 3 | 2 |
| *A. integra* | 3 | 2 |
| *A. rumphiana* | 0 | 0 |
| *A. alba* | 0 | 0 |
| *A. marina var. marina* | 2 | 1 |
| *A. marina var. eucarlyptifolia* | 1 | 1 |
| *A. marina var. australasica* | 1 | 1 |
| *A. germinans* | 3 | 2 |
